# Doxycycline Trap: A Case Report on the Unexpected Development of an Oesophageal Ulcer

**DOI:** 10.7759/cureus.71022

**Published:** 2024-10-07

**Authors:** Mladen Maksic, Tijana Veljkovic, Irfan Ćorović, Natasa Zdravkovic, Marina Jovanovic, Djordje Kralj, Dusan Radojevic, Djordje Stevanovic, Vojislav Djukic, Zeljko Todorovic, Biljana Milojkovic Kicevska

**Affiliations:** 1 Gastroenterology and Hepatology, University Clinical Center Kragujevac, Kragujevac, SRB; 2 Department of Internal Medicine, University of Kragujevac, Faculty of Medical Sciences, Kragujevac, SRB; 3 Department of Pediatrics, University of Kragujevac, Faculty of Medical Sciences, Kragujevac, SRB; 4 Department of Immunology and Microbiology, Center for Molecular Medicine and Stem Cell Research, Kragujevac, SRB; 5 Department of Internal Medicine, General Hospital of Novi Pazar, Novi Pazar, SRB; 6 Gastroenterology and Hepatology, KBC Zvezdara, Belgrade, SRB; 7 Department of Pharmacology and Toxicology, University of Kragujevac, Faculty of Medical Sciences, Kragujevac, SRB; 8 Gastroenterology, Clinic for gastroenterology and hepatology, Kragujevac, SRB

**Keywords:** chest pain, doxycycline, dysphagia, oesophageal ulcer, upper endoscopy

## Abstract

The phenomenon of drug-induced mucosal damage has not been reported much in the literature, although over 100 drugs are known to cause this condition. Tetracyclines contribute the most to mucosal damage. The common symptoms are difficulty swallowing, pain while swallowing, and possibly retrosternal pain. The diagnosis is based on upper endoscopy. The therapeutic approach involves discontinuing the causative drug, switching to parenteral fluid intake, and administering proton pump inhibitors and antacids. Proper drug intake in an upright position with plenty of fluids is key to preventing mucosal damage.

## Introduction

The mucosa of the oesophagus and stomach is exposed to several substances that can cause damage. Drug-induced mucosal damage has not been described often in the literature, although over 100 drugs are known to cause it [1]. The reason for the exacerbation of the damage is the neglect of symptoms, the underreporting of adverse effects of the therapy, and the lack of scientific publications addressing this issue. Tetracyclines and nonsteroidal anti-inflammatory drugs (NSAIDs), especially those in capsule form, contribute the most to mucosal damage, which gets aggravated when the drug is taken improperly [2]. The predominant symptoms are difficulty swallowing, pain during swallowing, and retrosternal pain. The onset of these symptoms can vary from a few hours to several days after ingestion. Diagnosis is made by performing an upper endoscopy [3].

This case report aims to highlight the rare adverse effect of doxycycline when taken incorrectly, as well as the significant beneficial effects of proton pump inhibitors, antacids, and hyaluronan in mucosal recovery within a short period.

## Case presentation

A 39-year-old woman presented to the emergency centre with retrosternal pain and difficulty and pain while swallowing. She reported that the pain was also present at rest and worsened when swallowing both liquids and solids. The symptoms began the day before her visit to the emergency centre. She mentioned that she had been taking doxycycline capsules, 100 mg twice daily, for the past 15 days. The medication was prescribed by a gynaecologist for a Ureaplasma urealyticum infection. She took the medication at 12 pm and 12 am, with the night dose taken without water while lying down. She denied having these symptoms previously. Furthermore, she denied weight loss, rectal bleeding, melena, or abdominal pain.

**Figure 1:**
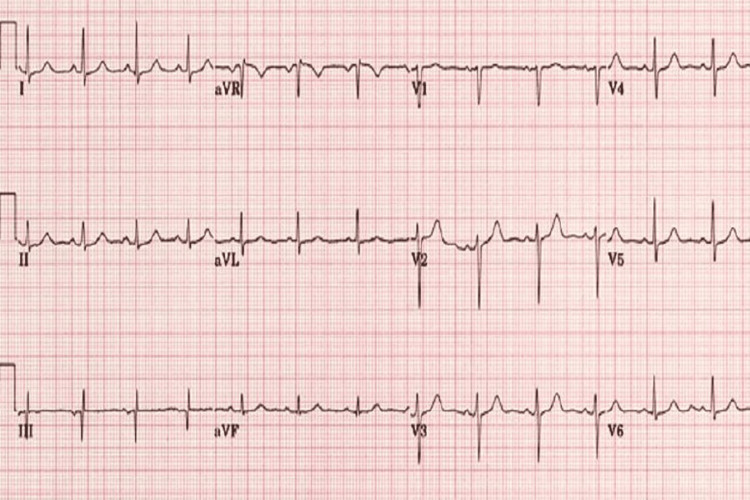
A normal electrocardiogram finding in the emergency department. Sinus rhythm, heart rate 90/min, no changes in ST and T segments.

In addition, the patient stated that she did not have any medication or food allergies and that she had not taken any therapy for chronic diseases. Also, she had no history of surgeries or tumours. She was a non-smoker and reported very rare alcohol consumption. The initial examination included anamnesis, a physical examination, an electrocardiogram (Figure 1), and laboratory tests (Table 1). Complete blood count and troponin levels were normal. The patient was referred to a gastroenterologist for further evaluation.

**Table 1:** Laboratory values. During hospitalization, the patient underwent the following laboratory analyses to assess overall health status and monitor the effectiveness of treatment. MCV: mean cell volume; TIBC: total iron-binding capacity; UIBC: unsaturated iron binding capacity; INR: international normalized ratio; aPTT: activated partial thromboplastin time; ALT: alanine aminotransferase; ALT: aspartate aminotransferase; GGT: gamma-glutamyltransferase; ALP: alkaline phosphatase; TSH: thyroid-stimulating hormone; FT4: free thyroxine test.

Variable	Patient analysis	Reference range, adults
White blood cells	4.5 × 10^9^/L	4.00–10.00 × 10^9^/L
Red blood cells	4.7 × 10^12^/L	4.34–5.72 × 10^12^/L
Platelets	161 × 10^9^/L	150–450 × 10^9^/L
Haemoglobin	140 g/L	138–175 g/L
MCV	85 fL	83.0–100.0 fL
Iron	14 μmol/L	14–32 μmol/L
TIBC	52 μmol/L	49–72 μmol/L
UIBC	33 μmol/L	23.45–76.08 μmol/L
Ferritin	31 μg/L	30–400 μg/L
Prothrombic time - INR	1.06	0.8–1.1
Prothrombin time	12.3 sec	11–13.5 sec
aPTT	32.9 sec	30–40 sec
Albumins	38 g/L	35–55 g/L
Urea	2 mmol/L	1.8–7.1 mmol/L
Creatinine	67 μmol/L	62–106 μmol/L
AST	12 U/L	10–36 U/L
ALT	13 U /L	5–38 U/L
GGT	31 U/L	5–36 U/L
ALP	46 U/L	44–147 U/L
Total bilirubin	14 μmol/L	1.71–20.5 μmol/L
Direct bilirubin	4 μmol/L	5.1 μmol/L
Natrium	137 mmol/L	135–145 mmol/l
Potasium	4.2 mmol/L	3.5–5.2 mmol/L
Troponin T	0.001 ng/mL	0.04 ng/mL
proBNP	124 pg/mL	0–125 pg/mL
C-reactive protein	0.7	0.3–1.0 mg/dL
TSH	2 mU/L	0.5–4.1 mU/L
FT4	1.6	0.7–1.9 ng/dL

**Figure 2:**
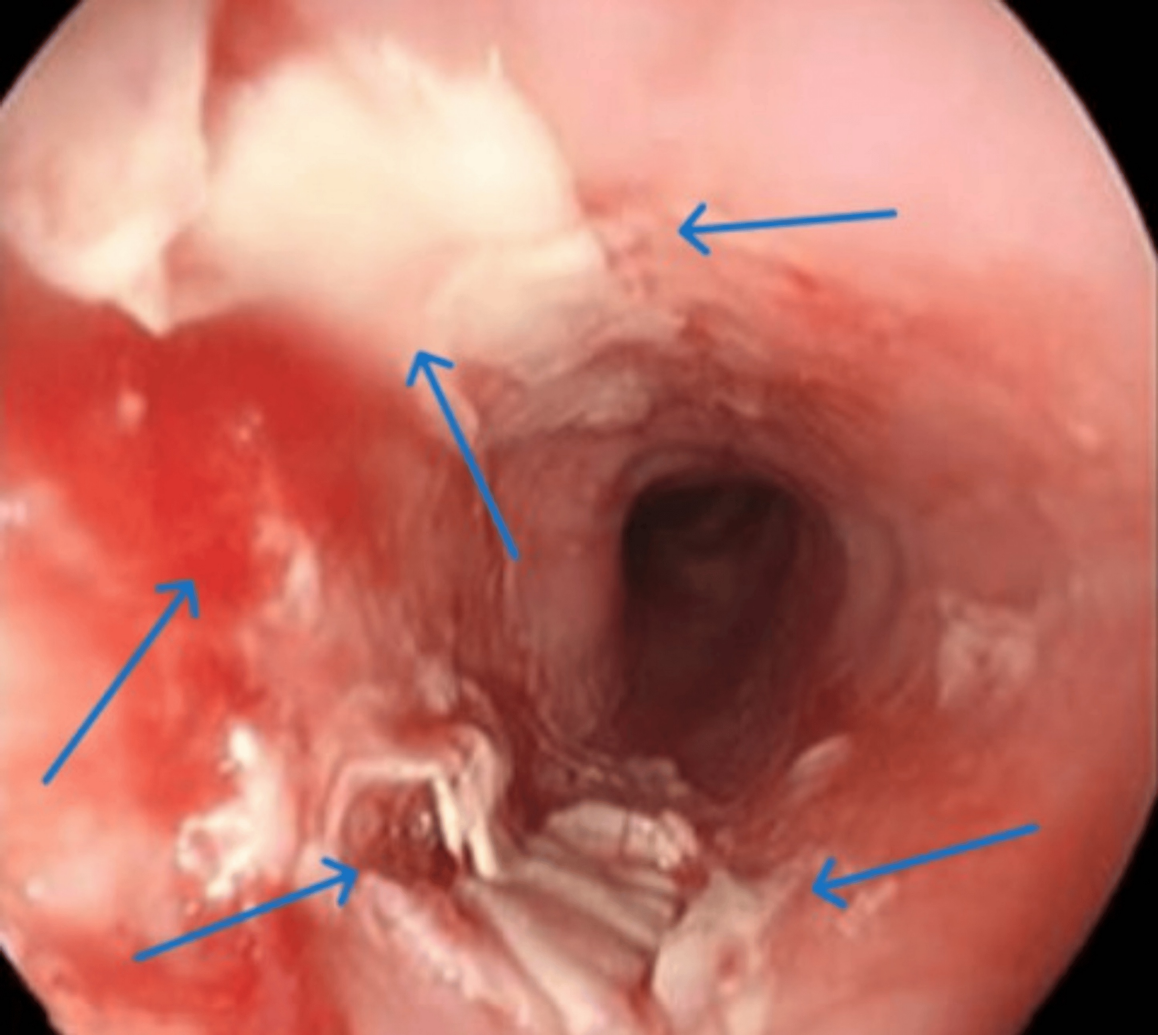
Multiple ulcerative lesions. Multiple ulcerative lesions (blue arrows) of varying sizes were identified approximately 30 cm from the incisors, with the larger ones measuring approximately 1.5 cm × 2 cm in diameter. The ulcers were shallow and had a broad base with a small amount of white plaque visible on their surface. The surrounding mucosa was raised, and approximately three-quarters of the cavity was affected.

Owing to dysphagia and odynophagia, an urgent gastroscopy was indicated by the gastroenterologist. During the procedure, multiple erosions and ulcer niches of the kissing ulcer type were observed in the thoracic region of the oesophagus, 30 cm from the incisors, and biopsy samples were acquired from the ulcer edges (Figure 2).

**Figure 3:**
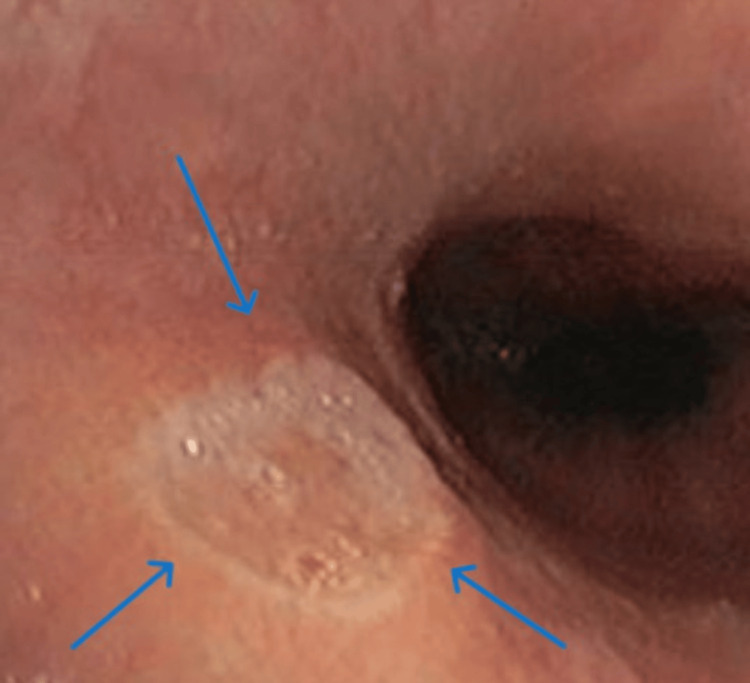
Findings on the oesophagus after the seventh day of therapy.

**Figure 4:**
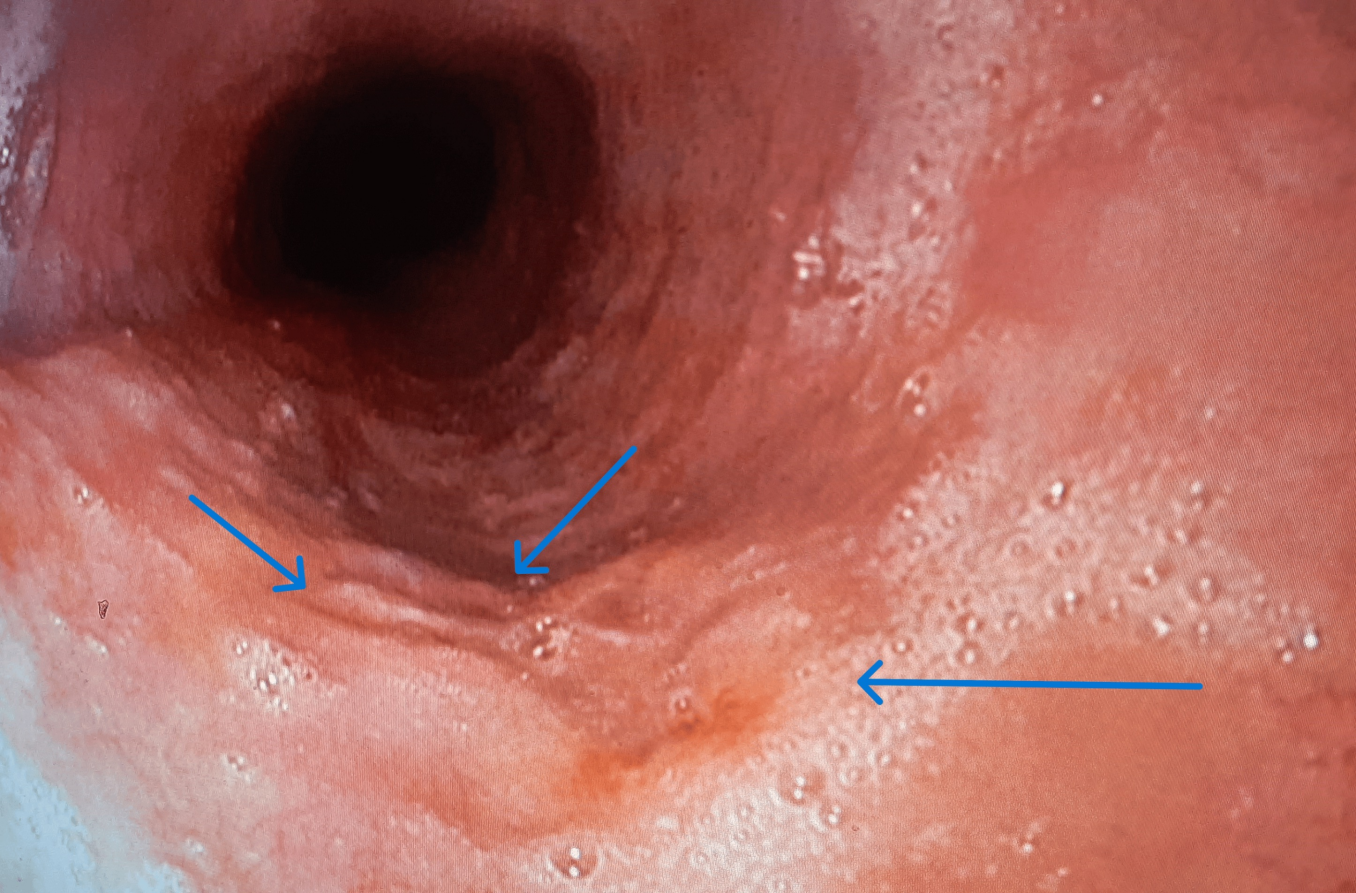
Findings on the oesophagus after the 14th day of therapy. The image shows a gastroscopic view of the stomach on the 14th day after the initiation of therapy. The previously documented ulcer niches are in the process of healing, with a noticeable reduction in their size and edges displaying signs of mucosal regeneration. No active bleeding is observed, and the surrounding mucosa appears relatively well preserved (blue arrows).

The rest of the gastroscopic findings were normal. Doxycycline was discontinued, and the patient was hospitalized for further treatment, including the administration of intravenous proton pump inhibitors and antacids, cessation of oral food intake, and transition to parenteral nutrition. The patient felt better two days after the initiation of therapy and discontinuation of doxycycline. A follow-up gastroscopy was performed 7 and 14 days later, which showed significant regression of the findings and symptomatology (Figures 3-4). The patient was discharged, and further home treatment was advised, with the recommendation to continue the following oral therapy: proton pump inhibitors (Rabezol®, AbelaPharm d.o.o.) 20 mg once a day for the next six weeks.

**Figure 5:**
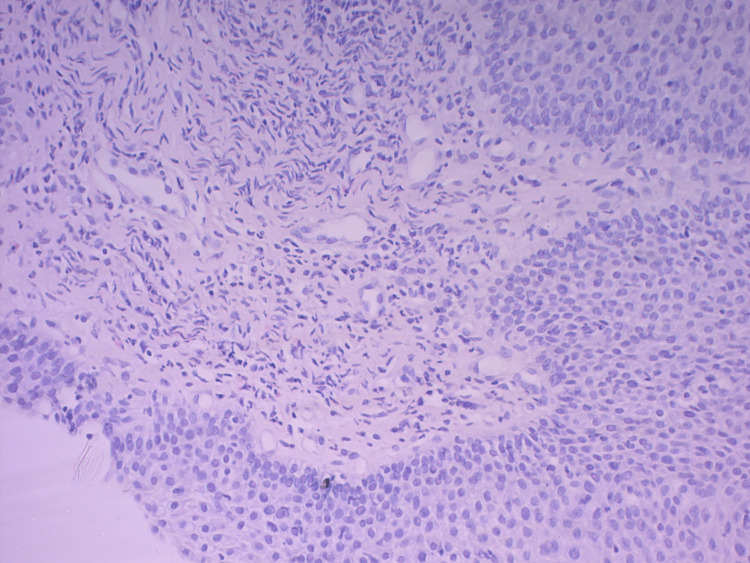
Histopathological section of the ulcer tissue. Haematoxylin and eosin staining showed acute and chronic inflammation of the squamous epithelial mucosa.

**Figure 6:**
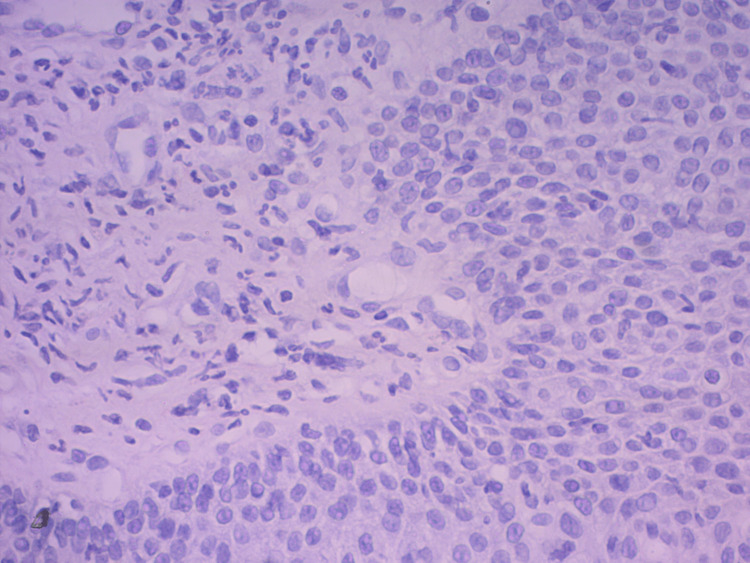
Histopathological section of the ulcer tissue. Haematoxylin and eosin staining showed acute and chronic inflammation of the squamous epithelial mucosa.

**Figure 7:**
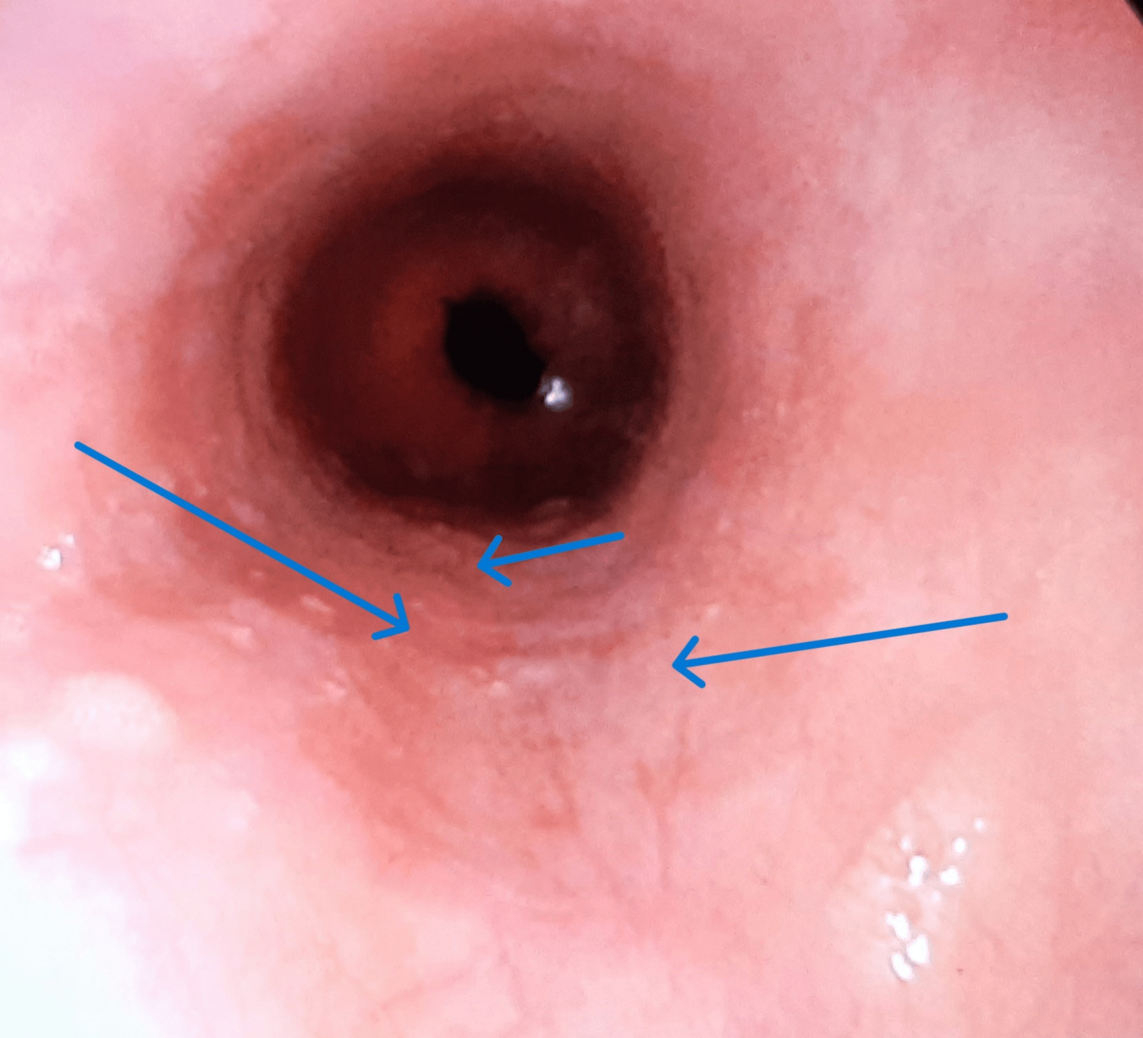
Findings on the oesophagus after the 21st day of therapy. The image shows a gastroscopic view of the stomach on the 21st day after the initiation of therapy. The previously documented ulcer niches have undergone complete healing, with the mucosal tissue fully regenerated and no visible signs of the previous lesions. The surrounding mucosa appears entirely healthy, with no active bleeding or scarring (blue arrows).

Histopathological findings showed that the squamous epithelium demonstrated signs of acute inflammation with the ulceration. Pronounced intramucosal leukocyte infiltration was observed, with a thin layer of subepithelial stroma whose blood vessels exhibited signs of leukocyte stasis (neutrophils and eosinophils) (Figures 5-6). A follow-up gastroscopy was performed 21 days after the patient’s discharge, which indicated complete mucosal healing and absolute cessation of dysphagia, odynophagia, and retrosternal pain (Figure 7).

## Discussion

The first case of mucosal damage in the upper digestive tract was described in 1970 [4]. The incidence is believed to be quite high, but it remains unrecognized as patients do not seek medical attention, and the symptoms resolve after discontinuing the drug. Some studies have reported that approximately 10,000 people in the USA experience mucosal damage in the oesophagus annually. Young individuals are not protected from adverse drug effects; on the contrary, the highest incidence occurs between 13 and 44 years of age. Although this damage occurs in both sexes, it is slightly more common in women [5].

The drugs that most frequently cause oesophagitis and oesophageal ulcers are tetracyclines, particularly doxycycline, followed by NSAIDs, potassium chloride and ferrous sulphate preparations, alendronate and quinidine. The primary mechanism is considered to be corrosion. The drug accumulates in the oesophageal mucosa, exerting a cytotoxic effect and inhibiting protein synthesis in the mucosa, which results in damage. This damage particularly occurs in the case of drugs taken in capsule form, where the gelatinous coating maintains longer contact with the mucosa than tablets [6].

Key risk factors for mucosal damage include previous oesophagitis, scleroderma, oesophageal motility disorders, and hiatal hernia. The method of drug intake also plays a crucial role. The medication should be taken with sufficient fluids, and an upright position should be maintained for at least 30 min. In our case, the patient took the medication at night while lying down and with little water. Another reason for mucosal damage is reduced saliva production and slowed oesophageal peristalsis at night [7].

According to the literature, symptoms typically arise within a few hours to 10 days. In our patient’s case, the symptoms appeared after two weeks of taking the medication. Numerous studies have examined the frequency of symptoms, with patients most commonly experiencing odynophagia (90% of the cases) and retrosternal pain (80%). Dysphagia is noted in only 50% of the cases [5]. In our case, the patient predominantly had dysphagia, which served as the decisive factor for performing an upper endoscopy.

In most cases, lesions are localized in the mid or thoracic portion of the oesophagus, which was also the case with our patient [8]. This localization is attributed to the anatomical impression of the aortic arch and the left atrium on the thoracic oesophagus [9].

During gastroscopy, several crescent-shaped ulcers covered with fibrin and containing inflamed and oedematous edges were observed. The rest of the oesophageal and gastric mucosa was normal. A biopsy from the edges of the lesions showed inflammatory and necrotic changes, with neutrophil and eosinophil infiltration.

The symptoms subsided after discontinuing doxycycline and administering proton pump inhibitors and antacids. Follow-up gastroscopies signified the resolution of the ulcers. The second gastroscopy, performed seven days after the first one, showed considerable improvement, and the third gastroscopy, done 14 days after discharge, demonstrated complete mucosal healing. The healing time of one to four weeks is aligned with the literature data [10].

## Conclusions

Adequate patient education on the proper intake of medications, especially those in the tetracycline group, is vital. The medication should be taken with at least 250 mL of water, and the patient should remain in an upright position for at least 30 min regardless of age, sex, or mucosal condition. Patients should be advised that if they experience symptoms such as difficulty swallowing, pain while swallowing or retrosternal pain, they should immediately discontinue the medication and consult a physician. Reporting adverse drug effects and publishing studies on this topic will raise awareness among physicians, pharmacists, and patients. By doing so, the occurrence of this problem could be prevented, diagnosis can be expedited if any adverse effect occurs, and life-threatening complications can be averted.
